# Degree centrality-based resting-state functional magnetic resonance imaging explores central mechanisms in lumbar disc herniation patients with chronic low back pain

**DOI:** 10.3389/fneur.2024.1370398

**Published:** 2024-06-11

**Authors:** Jianbing Mei, Yong Hu

**Affiliations:** Department of Radiology, Yongchuan Hospital of Chongqing Medical University, Chongqing, China

**Keywords:** lumbar disc herniation, chronic low back pain, functional neuroimaging, resting-state functional connectivity, degree centrality

## Abstract

**Objective:**

To investigate the central mechanism of lumbar disc herniation in patients with chronic low back pain (LDHCP) using resting-state functional magnetic resonance imaging (rs-fMRI) utilizing the Degree Centrality (DC) method.

**Methods:**

Twenty-five LDHCP and twenty-two healthy controls (HCs) were enrolled, and rs-fMRI data from their brains were collected. We compared whole-brain DC values between the LDHCP and HC groups, and examined correlations between DC values within the LDHCP group and the Visual Analogue Score (VAS), Oswestry Dysfunction Index (ODI), and disease duration. Diagnostic efficacy was evaluated using receiver operating characteristic (ROC) curve analysis.

**Results:**

LDHCP patients exhibited increased DC values in the bilateral cerebellum and brainstem, whereas decreased DC values were noted in the left middle temporal gyrus and right post-central gyrus when compared with HCs. The DC values of the left middle temporal gyrus were positively correlated with VAS (*r* = 0.416, *p* = 0.039) and ODI (*r* = 0.405, *p* = 0.045), whereas there was no correlation with disease duration (*p* > 0.05). Other brain regions showed no significant correlations with VAS, ODI, or disease duration (*p* > 0.05). Furthermore, the results obtained from ROC curve analysis demonstrated that the Area Under the Curve (AUC) for the left middle temporal gyrus was 0.929.

**Conclusion:**

The findings indicated local abnormalities in spontaneous neural activity and functional connectivity in the bilateral cerebellum, bilateral brainstem, left middle temporal gyrus, and right postcentral gyrus among LDHCP patients.

## 1 Introduction

Chronic low back pain (CLBP) is characterized by a musculoskeletal syndrome involving lumbar, sacral, and buttock pain or numbness persisting for more than 3 months. It is lasting, etiologically complex, and more prolonged than compared with acute pain (4–12 weeks) (1, 2), and represents one of the leading causes of disability worldwide, imposing a heavy burden on societies and individuals (3). Lumbar disc herniation (LDH) occurs due to a rupture of the annulus fibrosus of the intervertebral disc, leading to protrusion or prolapse of the nucleus pulposus into the posterior spinal canal. This results in irritation or compression of the corresponding nerve roots, manifesting as a series of clinical symptoms including CLBP (4, 5). LDH is one of the main causes of CLBP, resulting in physical inactivity, increased psychological distress, diminished social functional capacity, and a significant reduction in quality of life (6). Chronic pain is associated with both morphological and functional reorganization of the brain (7, 8), and lumbar disc herniation patients with chronic low back pain (LDHCP) exhibit disruptions in functional networks across the brain (9). LDH may cause compression or irritation of the spinal cord, potentially leading to damage of spinal cord neurons. This damage can be transmitted to the brain via transverse neural pathways, affecting brain function. Furthermore, LDH is linked to elevated levels of inflammatory factors (10), which may penetrate the brain via circulation, influencing neuronal activity and brain function. Consequently, differences in brain mechanisms between LDHCP and other causes of CLBP may exist, potentially related to the source of pain, neuropathology, and inflammatory response. Given its high prevalence and significant contribution to disability, understanding the functional properties of the brain in LDHCP patients is of paramount importance.

In recent years, the use of resting-state functional magnetic resonance imaging (rs-fMRI), a noninvasive neuroimaging technique (11), to study neural activity in the brains of CLBP patients has garnered increasing attention. Hong Li chose the thalamus as the seed for resting-state functional connectivity analysis and found abnormal alterations in brain function between the thalamus and the dorsolateral prefrontal cortex (DLPFC) in patients with LDHCP (12). Zhang used the amplitude of low-frequency fluctuation (ALFF) method to find abnormal alterations in brain function in the precentral gyrus, paracentral lobule, and para hypophyseal motor areas of patients with CLBP (13). Fuqing Zhou used the Regional Homogeneity (ReHo) method to find abnormal alterations in brain function in the posterior lobe of the right cerebellum, brainstem, left medial prefrontal cortex, and bilateral precuneus in patients with LDHCP (14). Previous studies on brain function in CLBP patients have concentrated on functional connectivity, ALFF, and ReHo. However, metrics that provide information on whole-brain functional connectivity (e.g., based on seed points) necessitate *a priori* assumptions that could bias the results. Moreover, ALFF and ReHo do not capture alterations in whole-brain functional connectivity. Degree centrality (DC) is an important component of graph theory and network analysis, which can reflect the strength of functional connectivity across the brain without *a priori* assumptions, helping to determine the influence of specific brain regions on pain processing and revealing how connectivity between these brain regions changes in LDHCP patients. The larger the DC value, the more brain regions are connected to the node of interest. Among several large-scale network metrics, DC is considered the most reliable (15) and has been widely used in brain network research (16–18). Additionally, there is a paucity of functional magnetic resonance imaging (fMRI) research on the brain function of individuals within the LDHCP population. Consequently, this study will employ DC analysis to thoroughly investigate functional brain changes in LDHCP and examine the correlations with the Visual Analogue Score (VAS), Oswestry Dysfunction Index (ODI), and disease duration.

## 2 Materials and methods

### 2.1 Subjects

This prospective study received approval from the Ethics Committee of Yongchuan Hospital of Chongqing Medical University (Approval No. 2022–72, Date: 2022-06-30). Written informed consent was obtained from all participants, and the methods were conducted in accordance with the approved guidelines. A total of 27 LDHCP outpatients (LDHCP group) and 22 age-and gender-matched healthy volunteers (HC group) were recruited from Yongchuan Hospital of Chongqing Medical University between August 2022 and August 2023. Prior to the MRI examination, LDHCP outpatients completed a questionnaire regarding their current pain, including disease duration, Visual Analogue Score (VAS), and Oswestry Dysfunction Index (ODI) questionnaire scale.

Inclusion criteria for the LDHCP group were as follows: (1) right-handedness; (2) LDH confirmed by clinical presentation, physical examination, and lumbar spine CT or MRI; (3) pain duration was longer than 3 months and pain intensity was 3 or higher on a 0–10 VAS during screening; (4) no use of antipyretic-analgesic, sleeping, or hormonal medications within 1 week; (5) between the ages of 18 and 60, regardless of gender; (6) no pain other than LDH-related pain; (7) no psychiatric or neurological disorders; and (8) no abnormal findings such as infarcts or focal lesions on brain MRI presentation confirmed by two uninformed independent radiologists.

Inclusion criteria for the HC group were as follows: (1) right-handedness; (2) between the ages of 18 and 60, regardless of gender; (3) no history of CLBP or other chronic pain conditions; (4) general health with no history of chronic systemic diseases, such as diabetes mellitus or hypertension; (5) no psychiatric or neurological disorders; and (6) absence of abnormal findings, such as infarcts or focal lesions, on brain MRI manifestations, as confirmed by two uninformed independent radiologists.

Exclusion criteria for the LDHCP and HC groups were as follows: (1) contraindications to and intolerance of MRI examination, such as claustrophobia or metallic implants; (2) suffering from other chronic pain disorders; (3) other abnormal signal changes in the brain parenchyma detected by routine MRI; (4) history of psychiatric disorders; (5) age < 18 or > 60 years; and (6) history of surgery or lumbar vertebral fracture or neurological disorders.

### 2.2 Instruments and methods

MRI scans were performed on a 3.0 T Siemens Magnetom Verio scanner (Siemens Healthcare, Erlangen, Germany) using a 12-channel phased-array head coil. Subjects underwent conventional T2-weighted imaging (T2WI), three-dimensional T1-weighted imaging (3D-T1WI) and rs-fMRI. To minimize head movement and reduce noise interference, a foam pad, antimagnetic earplugs, and cotton balls were used. Participants were instructed to stay awake, breathe steadily, relax with their eyes open, and refrain from engaging in specific thought processes throughout the test.

3D-T1WI scan: A three-dimensional magnetization-prepared gradient rapid acquisition gradient echo (MPRAGE) sequence was used to obtain sagittal T1WI, which included the whole brain and can provide a template for the segmentation of gray matter, white matter, and cerebrospinal fluid. The scanning parameters were as follows: repetition time (TR) = 2,300 ms, echo time (TE) = 2.27 ms, field of vision (FOV) = 256 × 256 mm, scanning slices = 192 layers, slice thickness = l mm, slice gap = 0 mm, flip angle = 7°, voxel size = 1 × 1 × 1 mm3.

Rs-fMRI scan: The gradient echo-plane echo imaging pulse sequence was used, and 240 time points were acquired in this sequence for functional data acquisition. The scanning parameters were as follows: TE = 30 ms, TR = 2,000 ms, scanning slices = 35 layers, slice thickness = 4 mm, slice gap = 0.5 mm, FOV = 216 mm × 216 mm, flip angle = 90°.

### 2.3 Image analysis

Image preprocessing was conducted using the Data Processing & Analysis for Brain Imaging (DPABI)^1^ (19) and SPM12^2^ running on MATLAB R2018b.^3^ The preprocessing steps were: (1) format conversion; (2) removal of the data of the first 10 time points (volume) to reduce or eliminate the effect of these data on the results; (3) temporal layer correction; (4) head-motion correction, which removes subject data with translation >3 mm or rotation >3° in either direction; (5) alignment using the MPRAGE sequence of images to normalize brain Functional images were aligned using MPRAGE sequence images, normalized to the Montreal neurological institute (MNI) standard template, and resampled with 3 mm × 3 mm × 3 mm voxels; (6) linear offset was removed; (7) regression covariates included cerebral white matter, cerebrospinal fluid signals, and Friston’s 24 cephalic motion parameters; (8) filtering was performed to retain the signal at 0.01–0.1 Hz.

Voxel DC computation was performed with the DPABI package by treating each voxel as a node. This involved extracting the time series for each voxel, calculating the Pearson correlation coefficient between the time series of each voxel and all other voxels, and setting the threshold of the obtained Pearson correlation coefficient matrix to the classical reference value of 0.25 (20). Binary DC values were employed (21, 22), and Fisher-Z transformation was applied to the DC values of each voxel to generate a z-plot of the gray matter DC values for each subject. Image smoothing during statistical analysis was achieved using a Gaussian kernel function with a half-height width of 6 mm.

### 2.4 Statistical analysis

Statistical analyses were conducted using the Statistical Product and Service Solution (SPSS) software version 27.0 (IBM, Armonk, NY, United States). Categorical variables were presented as n (%), and the chi-square test was utilized to compare DC values between patients and HCs. The Kolmogorov–Smirnov test was employed to assess the normality of continuous quantitative data such as age and clinical scores. Normally distributed measurements were expressed as mean ± standard deviation (SD), and two independent samples *t*-tests were conducted to compare differences in DC values. Non-normally distributed measurements were expressed as median (interquartile range). Pearson and Spearman correlation analyses were used to examine correlations between DC values in different brain regions and VAS, ODI, and disease duration. *p* < 0.05 was considered statistically significant. The Gaussian random field (GRF) method was used to correct for multiple comparisons (two-tailed, voxel-level *p* < 0.001; GRF correction, cluster-level *p* < 0.05). Receiver operating characteristic (ROC) curves of subjects with differential brain region DC values were plotted to analyze the sensitivity and specificity of significant differential brain regions in distinguishing between LDHCP and HCs.

## 3 Results

### 3.1 Demographic and clinical data

**Figure 1:**
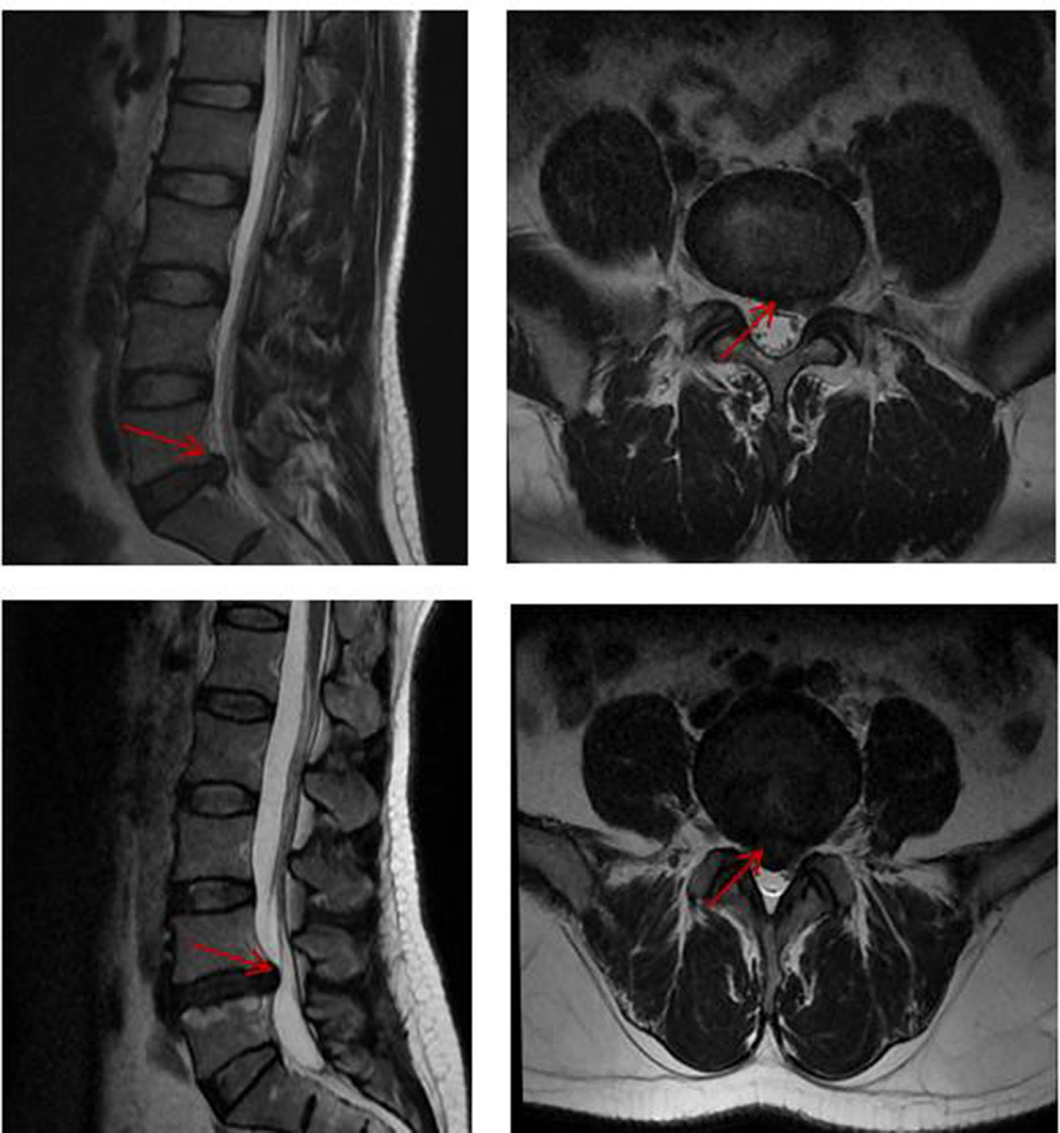
Lumbar spine MRI in some patients in the LDHCP group. Red arrows represent herniated discs.

The demographic and clinical features of the enrolled LDHCP and the HC groups are listed in Table 1. Two patients in the LDHCP group were excluded because of excessive head motion. A total of 25 LDHCPs and 22 HCs were finally included. There was no statistically significant difference in the age and gender of the subjects between the two groups (*p* > 0.05). And Figure 1 demonstrates lumbar spine MRI in some patients in the LDHCP group.

**Table 1:** Demographic characteristics of the LDHCP group and HC group.

Group	Age	Gender		Disease duration (year)	VAS	ODI
		Male	Female			
LDHCP group (*n* = 25)	35.40 ± 10.96	12 (48.0%)	13 (52.0%)	3.0 (4.8)	5.32 ± 1.65	0.25 ± 0.15
HC group (*n* = 22)	31.45 ± 9.63	14 (63.6%)	8 (36.4%)	–	–	–
X^2^/t	−1.466	1.158		–	–	–
P	0.15	0.282		–	–	–

### 3.2 DC value results

**Figure 2:**
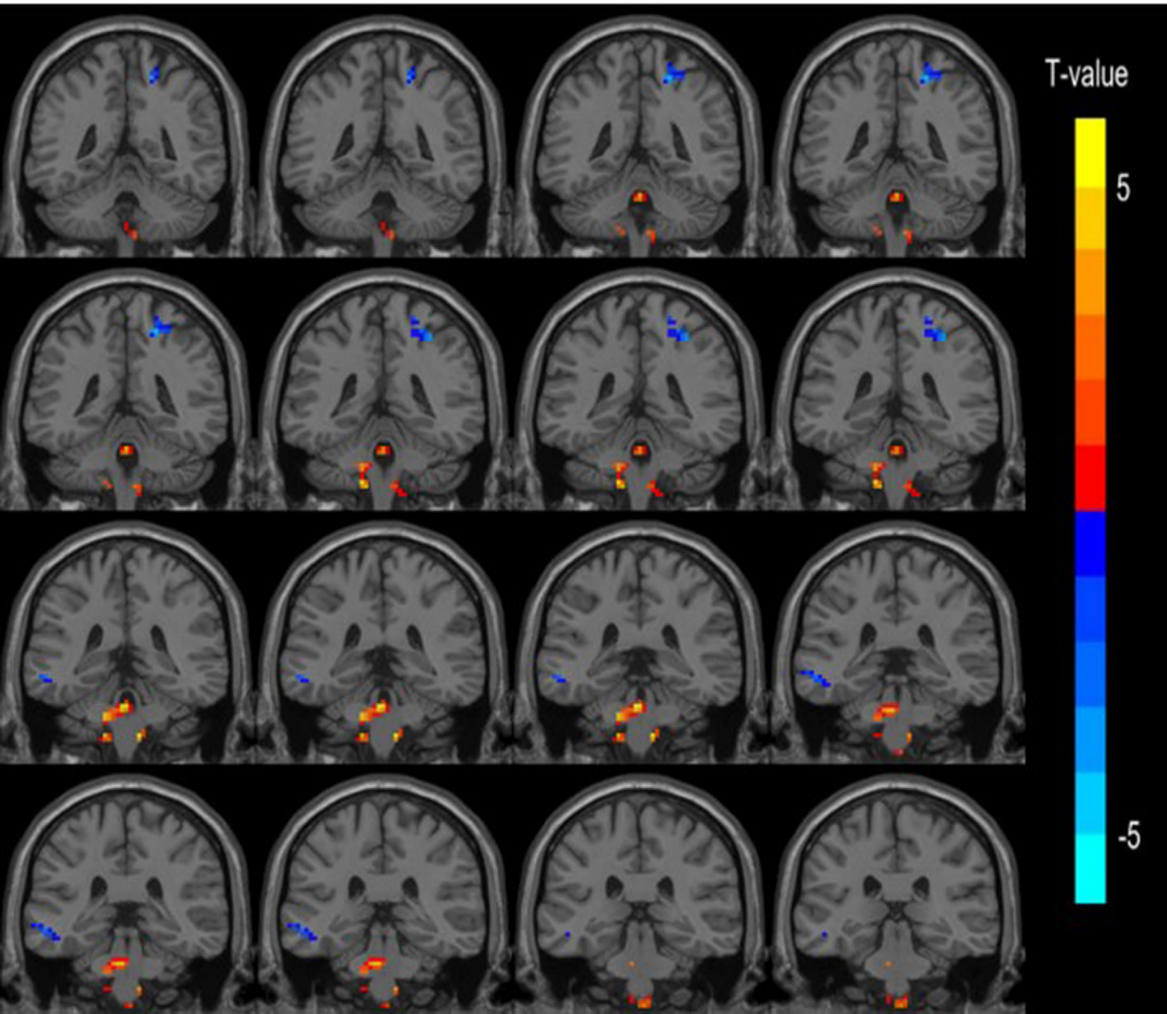
Spatial location map of abnormally activated brain regions in the LDHCP.

Compared with the HC group, the cerebellar and brainstem DC values were increased bilaterally in the LDHCP group; the DC values were decreased in the left middle temporal gyrus and the right postcentral gyrus, and the differences were statistically significant (two-tailed, voxel-level *p* < 0.001; GRF correction, cluster-level *p* < 0.05). The difference brain regions are shown in Table 2 and Figure 2.

**Table 2:** Brain region with a significant difference in DC value between the LDHCP group and HC group.

Brain regions	Cluster size (voxels)	Peak MNI coordinates			Peak point *t*-value
		x	y	z	
Brainstem_L, Cerebellum_L	50	−3	−36	−30	4.35392
Brainstem_R, Cerebellum_R	44	9	−36	−51	4.4352
Temporal_Mid_L	39	−51	−24	−15	−4.69497
Postcentral_R	35	21	−42	57	−5.02707

Two-sample *t*-test results of DC value between the LDHCP and HC groups (two-tailed, voxel-level *P* < 0.001; GRF correction, cluster-level *P* < 0.05).

### 3.3 Clinical magnetic resonance imaging correlations

**Figure 3:**
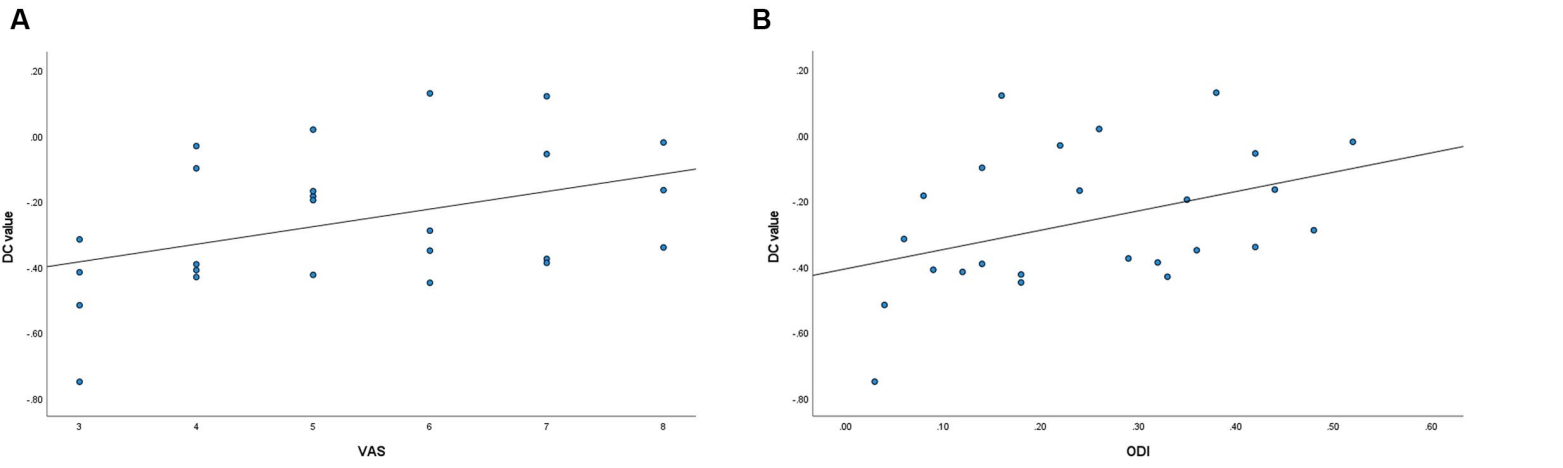
**(A)** Correlation between the left middle temporal gyrus DC values and VAS (*r* = 0.416, *p* = 0.039). **(B)** Correlation between the left middle temporal gyrus DC values and ODI (*r* = 0.405, *p* = 0.045).

The DC values of the left middle temporal gyrus in the LDHCP group showed a positive correlation with the VAS (*r* = 0.416, *p* = 0.039) and ODI (*r* = 0.405, *p* = 0.045), but no significant correlation with disease duration (*p* > 0.05). In addition, the DC values of the remaining brain regions had no correlation with VAS, ODI, and disease duration (*p* > 0.05) (Figures 3A,B).

### 3.4 ROC curve analysis

**Figure 4:**
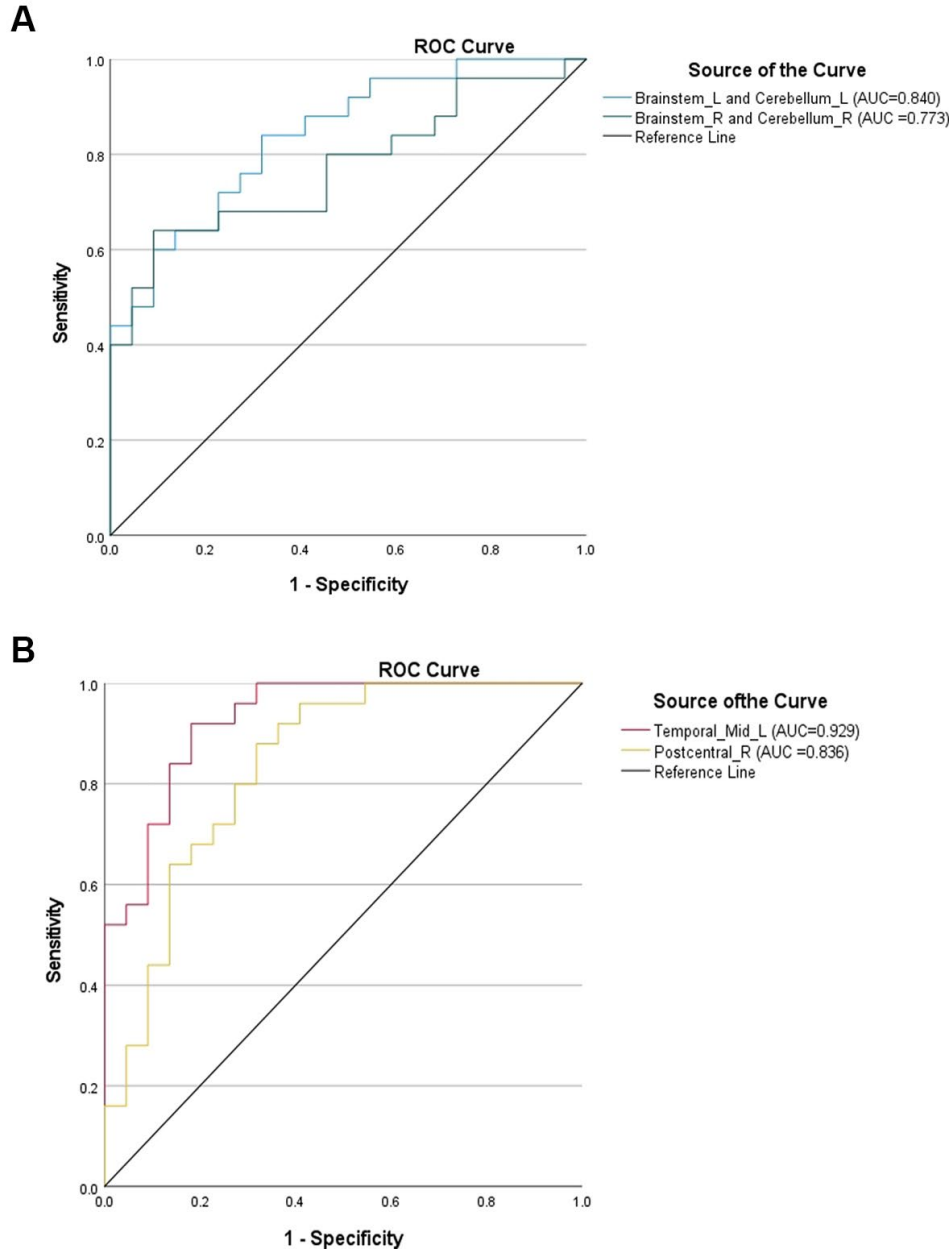
**(A)** ROC curves using brain regions with elevated DC values in the LDHCP group as discriminatory markers. **(B)** ROC curves using brain regions with reduced DC values in the LDHCP group as differentiating markers.

The DC values of the brain regions with differences between the LDHCP and HC groups were extracted, and the ROC curves were plotted to verify the diagnostic efficacy of the DC values of the different brain regions. The results show the area under the curve (AUC): left brainstem and left cerebellum (0.840, 95% CI: 0.730–0.950, *P* < 0.001), right brainstem and right cerebellum (0.773, 95% CI: 0.638–0.908, *P* < 0.001), left middle temporal gyrus (0.929, 95%CI:0.859–1.000, *P* < 0.001), and right posterior central gyrus (0.836, 95%CI:0.718–0.955, *P* < 0.001) (Figures 4A,B).

## 4 Discussion

The current study yielded three main observations: (1) the LDHCP group demonstrated increased DC values within the bilateral cerebellum and brainstem regions, alongside decreased DC values in the left middle temporal gyrus and right postcentral gyrus, compared to HCs. (2) DC values in the left middle temporal gyrus were positively correlated with the VAS and ODI, with no correlation observed concerning disease duration. Moreover, DC values in other brain regions did not correlate with VAS, ODI, or disease duration. (3) ROC curve analysis revealed high diagnostic efficacy of the left middle temporal gyrus.

The cerebellum, a crucial anatomical structure situated between the brainstem and cerebral hemispheres, plays a pivotal role in coordinating and regulating various functions, including movement, balance, and select cognitive processes such as emotional processing, learning, memory, and pain regulation (23–28). Animal studies have indicated that both electrical and chemical stimulation of the cerebellum can modulate nociceptive responses (29, 30). Furthermore, studies have consistently shown that cerebellar activation in both acute and chronic pain scenarios (31–33). Cerebellar lesions affect pain perception in humans, leading to enhanced nociception and diminished analgesia (34). In this study, elevated DC in the bilateral cerebellum of LDHCP patients suggests increased connectivity with other brain regions, potentially related to pain processing, modulation, or other neural mechanisms associated with pain. It is conceivable that LDHCP brains may employ compensatory mechanisms in response to chronic pain, with the cerebellum assuming a crucial role in this compensatory process. This could involve maintaining body balance and controlling movement to adapt to the effects of pain. Additionally, certain pain conditions may induce structural and functional brain changes, with the observed increased DC values in the cerebellum possibly reflecting abnormal remodeling in response to pain-related alterations.

The brainstem, a fundamental component of the central nervous system, oversees numerous vital physiological functions and neuromodulatory processes. It comprises regions capable of processing incremental nociceptive information and modulating neurotransmission at primary nociceptive synapses, both directly and indirectly (35, 36). Preclinical and human studies on brainstem pain modulatory circuits have elucidated the pain-modulatory functions of several key brainstem structures (2, 37, 38). This study’s observation of increased DC values in the brainstem among LDHCP patients further supports the brainstem’s role as a crucial neuromodulatory hub in transmitting and regulating pain signals. Additionally, elevated DC values in the brainstem may relate to increased inflammation and neuroimmune responses. Chronic pain states can provoke inflammatory factor release and immune response activation (39), impacting the brainstem and resulting in elevated DC values. Persistent chronic pain may also induce neuroplastic changes, including increased excitatory synaptic transmission and reduced inhibitory synaptic transmission (40). These neuroplastic changes within the brainstem, a critical node in neural signaling, can contribute to increased DC values. Furthermore, LDHCP may undergo changes in central sensitization, potentially leading to abnormal pain signal amplification or transmission at the brainstem level, and an increase in brainstem DC values is one way in which the central nervous system adapts to the state of pain.

The left middle temporal gyrus is instrumental in various cognitive domains, including language, hearing, vision, memory, and emotion. This region collaborates with other brain regions to form a complex neural network that is foundational to higher cognitive functions in humans. Studies have shown that temporal lobe involvement is associated with unpleasant emotions (41–43) and that the temporal lobe is primarily responsible for pain perception and emotional processing (5, 44, 45). Presently, researchers are observing structural and functional changes in this region in a variety of pain-related disorders (46–52). In our study, we discovered a reduction in the DC value of LDHCP in the left middle temporal gyrus, indicating potential difficulties in regulating pain and emotions; such dysregulation could lead to increased pain or emotional instability. This result may also relate to abnormal neural activity in this brain region, suggesting that the patient’s brain is attempting to adjust to the effects of pain. It is noteworthy that these findings may contribute to the development of more targeted treatment strategies.

The postcentral gyrus, a pivotal region of the cerebral cortex, is recognized for its significant contribution to cognitive control, emotion regulation, and information processing, especially in relation to the default mode network (DMN) (53, 54). Our study identified a reduction in the DC value of LDHCP within the right postcentral gyrus, suggesting a potential disruption in neural network connectivity in this area and potentially leading to cognitive and affective dysfunctions. Additionally, the postcentral gyrus is reputed for its role in the pain inhibition process (55, 56). Thus, diminished DC values in this region imply a compromised ability in pain modulation, which could influence pain perception and management in LDHCP, hinting at novel neural mechanisms underlying pain experiences. Moreover, these reduced DC values might be indicative of the phenomenon of pain chronicity, implying that the neural circuitry in the right postcentral gyrus undergoes adaptive changes and remodeling in response to prolonged pain conditions, which may be one of the potential mechanisms for chronicity in pain perception in patients.

Correlation analysis in our study unveiled a positive association between the DC value of the left middle temporal gyrus and both VAS and ODI scores in the LDHCP group. This suggests that the left middle temporal gyrus may have a distinctive neuroanatomical function in pain transmission and processing in LDHCP patients, contributing to pain perception and severity of the condition. The absence of a correlation between the DC values of the left middle temporal gyrus and disease duration in the LDHCP group could stem from individual clinical variations, such as diverse treatment histories among patients, potentially obscuring any direct association with disease duration. These findings underscore the need for additional research to elucidate the physiological and pathological mechanisms at play. Furthermore, while functional connectivity abnormalities were also present in other brain regions, they did not correlate with VAS, ODI, or disease duration. This suggests that various brain regions may have distinct roles in sustaining LDHCP, a condition characterized by a complex interplay of physical, psychological, and social elements. The results from ROC curve analysis, demonstrating an AUC of 0.929 for the left middle temporal gyrus, underscore a significant differentiation between the LDHCP and HC groups. This leads us to posit that the DC value of the left middle temporal gyrus may serve as a potential objective diagnostic marker for LDHCP.

From a clinical perspective, our study provides new perspectives on the treatment of LDHCP. First of all, by gaining a deeper understanding of the neurobiological hallmarks of the brain in LDHCP patients, we can target these abnormalities for more targeted treatment. For example, noninvasive brain stimulation techniques can be used to modulate the excitability of functional brain regions, target brain-derived neurotrophic factors to modulate neuroplasticity, and apply traditional rehabilitation therapies to regulate functional brain activity. In addition, our study provides new methods for the early diagnosis of LDHCP and prediction of patient outcomes, which can help clinicians detect lesions earlier and take appropriate therapeutic measures for better management and treatment of patients with LDHCP.

It is worth mentioning that in capturing subtle differences in the brain networks of LDHCP patients, DC can complement other network topological indicators, thus providing a more comprehensive understanding. Firstly, DC evaluates the number of aberrant functional connectivity in the brain network, which provides basic information about the importance of nodes in the network but overlooks the influence of nodes on information transmission or their position in the entire network. In comparison, global indicators such as clustering coefficient (Cp), characteristic path length (Lp), global efficiency (Eg), and small-world attribute (σ) offer a more holistic view of network characteristics (57). Cp reflects the degree of connectivity between nodes in the network, Lp evaluates the information transmission capacity of the network, Eg assesses the network’s ability to globally transmit information, and σ quantifies the network’s small-world characteristics. These indicators help capture changes in the overall structure of brain networks in LDHCP patients, providing a more comprehensive understanding. In addition, local indicators such as node betweenness and node efficiency can help reveal the importance and influence of individual nodes in information transmission. Node betweenness reflects the contribution rate of nodes to information exchange with other nodes, whereas node efficiency reflects the ability of nodes to propagate information to other network nodes. These indicators can help capture key nodes with significant effects in the brain networks of LDHCP patients, revealing subtle differences in the network. Therefore, future research should consider integrating degree centrality with other global and local indicators to comprehensively analyze changes in the brain networks of LDHCP patients, leading to a deeper understanding of the neural mechanisms underlying pain and related network alterations.

It is crucial to recognize the limitations inherent in this study. Firstly, being a single-center cross-sectional study with a relatively small participant base limits our ability to establish a direct causal link between the observed neural patterns and LDHCP. Future research should aim to conduct longitudinal studies with larger sample sizes to provide a more definitive exploration of these relationships. Secondly, in this study, only DC was analyzed, after which more network topology metrics will be included to characterize the changes in the functional networks of LDHCP patients from multiple perspectives. Thirdly, our study lacked an assessment of cognitive function and neuropsychological aspects of LDHCP. Prolonged low back pain can adversely impact patients’ emotional well-being, leading to symptoms such as irritability, anxiety, and depression. To gain a more holistic understanding of CLBP, future investigations should incorporate relevant clinical assessment tools to accurately evaluate the psychological status of patients. Fourthly, the focus was exclusively on brain activity, overlooking the assessment of clinical pain-related inflammatory markers. An integrative approach examining both neural and inflammatory indicators in future studies could illuminate the complex biological and neurophysiological dynamics involved. Lastly, while this study validated the diagnostic efficacy of different brain region DC values for LDHCP using ROC curves, we recognize that in actual clinical practice, the definitive diagnosis of LDHCP relies primarily on clinical presentation and lumbar MRI/CT imaging. Therefore, the clinical significance of using brain function indicators as new neurobiological markers is relatively limited.

## 5 Conclusion

This study illuminated localized disruptions in spontaneous neural activity and functional connectivity in the bilateral cerebellum, bilateral brainstem, left middle temporal gyrus, and right postcentral gyrus in LDHCP. These abnormally connected brain regions may be potential neurobiological markers of LDHCP and are expected to offer a fresh perspective on the treatment of LDHCP.

## Data availability statement

The original contributions presented in the study are included in the article/supplementary material, further inquiries can be directed to the corresponding author.

## Ethics statement

The studies involving humans were approved by the Ethics Committee of Yongchuan Hospital of Chongqing Medical University (No. 2022–72, Date: 2022-06-30). The studies were conducted in accordance with the local legislation and institutional requirements. The participants provided their written informed consent to participate in this study. Written informed consent was obtained from the individual(s) for the publication of any potentially identifiable images or data included in this article.

## Author contributions

JM: Writing – review & editing, Writing – original draft, Validation, Methodology, Investigation, Formal analysis, Data curation, Conceptualization. YH: Writing – review & editing, Validation, Supervision, Conceptualization.

## Footnotes

^1^http://rfmri.org/dpabi

^2^fil.Ion.ucl.ac.uk/spm

^3^https://uk.mathworks.com/products/matlab
